# Inhibition of the NLRP3 inflammasome using MCC950 reduces vincristine‐induced adverse effects in an acute lymphoblastic leukemia patient‐derived xenograft model

**DOI:** 10.1002/hem3.70092

**Published:** 2025-03-18

**Authors:** Hana Starobova, Hannah McCalmont, Svetlana Shatunova, Nicolette Tay, Christopher M. Smith, Avril Robertson, Ingrid Winkler, Richard B. Lock, Irina Vetter

**Affiliations:** ^1^ Institute for Molecular Bioscience, The University of Queensland St Lucia Queensland Australia; ^2^ Children's Cancer Institute, Lowy Cancer Research Centre, School of Clinical Medicine, UNSW Medicine & Health, UNSW Centre for Childhood Cancer Research, UNSW Sydney Sydney New South Wales Australia; ^3^ Mater Research Institute, The University of Queensland South Brisbane Queensland Australia; ^4^ School of Chemistry and Molecular Biosciences The University of Queensland St Lucia Queensland Australia; ^5^ School of Pharmacy and Pharmaceutical Sciences The University of Queensland Woolloongabba Queensland Australia

## Abstract

Vincristine is one of the most important chemotherapeutic drugs used to treat acute lymphoblastic leukemia (ALL). Unfortunately, vincristine often causes severe adverse effects, including sensory–motor neuropathies, weight loss, and overall decreased well‐being, that are difficult to control and that decrease the quality of life and survival of patients. Recent studies demonstrate that sensory–motor adverse effects of vincristine are driven by neuroinflammatory processes, including the activation of the Nod‐like receptor 3 (NLRP3) inflammasome. In this study, we aimed to test the effects of MCC950, a specific NLRP3 inhibitor, on the prevention of vincristine‐induced adverse effects as well as tumor progression and vincristine efficacy in NOD/SCID/interleukin‐2 receptor *γ*‐negative mice patient‐derived xenografts of ALL. We demonstrate that co‐administration of MCC950 effectively prevented the development of mechanical allodynia, motor impairment, and weight loss and significantly improved the overall well‐being of the animals without negatively impacting the *in vivo* efficacy of vincristine as a single agent or in combination with standard‐of‐care drugs. These results provide proof of principle that the adverse effects of vincristine chemotherapy can be prevented using NLRP3 inflammasome inhibitors and provide new options for the development of effective treatment strategies.

## Graphical abstract

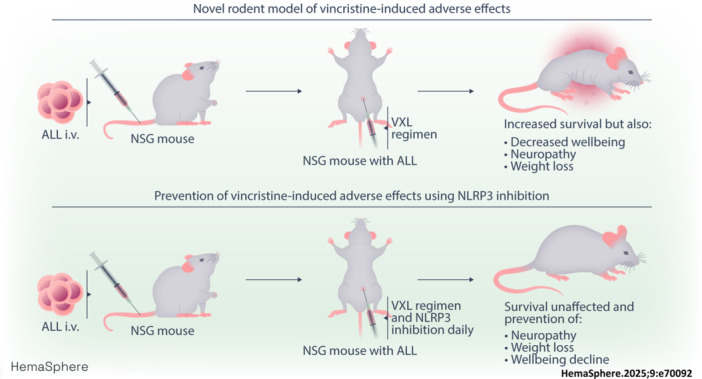

## INTRODUCTION

Blood cancers, including acute lymphoblastic leukemia (ALL), are the most frequently diagnosed cancers in children and are typically treated with combination chemotherapy regimens that include the use of vincristine together with, for example, *L*‐asparaginase and dexamethasone.^1^ Unfortunately, many pediatric patients treated with such chemotherapy regimens develop untreatable adverse effects that include sensory–motor neuropathy, weight loss, and an overall decrease in well‐being and quality of life (reviewed in Tay et al.^2^). For example, Lavoie Smith et al. reported that in pediatric ALL patients, vincristine causes grades 1, 2, and 3 sensory neuropathies with a prevalence of 31%, 3.2%, and 1.6%, respectively, and motor neuropathies in 18%, 4.4%, and 1.9%, respectively.^3^ The symptoms include paresthesia, neuropathic pain, numbness, and impairment in strength to name a few.^3^ Such adverse effects can be challenging to control, with current guidelines primarily recommending dose reduction or discontinuation of chemotherapy, an approach that can adversely affect the survival of pediatric patients.^4^

The reported prevalence of vincristine‐induced peripheral neuropathy (VIPN) in children reaches 0%–90%, suggesting it is multifactorial in origin (reviewed in Tay et al.^2^). Indeed, evidence suggests that VIPN mechanisms may include microtubule disruption, impaired axonal transport, axonal degeneration, and the disruption of mitochondrial function. Additionally, recent evidence from rodent studies provides a strong case for VIPN being driven by neuroinflammatory mechanisms.^5^, ^6^, ^7^, ^8^ Specifically, vincristine increases the number of F4/80^+^ cells in peripheral nervous tissue, such as the dorsal root ganglia and sciatic nerve.^9^, ^10^ Additionally, vincristine activates the Nod‐like receptor 3 (NLRP3) inflammasome in monocytes/macrophages, leading to the activation of caspase‐1 (CASP1) and the subsequent release of the nerve‐sensitizing interleukin‐1β (IL‐1β).^9^, ^11^ Consequently, inhibition of NLRP3 or the interleukin‐1 receptor (IL‐1R) fully prevents the development of vincristine‐induced sensory neuropathy in mice.^9^

However, previous animal studies, including those performed by us, that investigated the role of monocytes and NLRP3 signaling in VIPN utilized models that were based on the administration of vincristine only.^9^, ^10^, ^12^ Vincristine is usually administered in combination with other drugs, such as glucocorticoids,^13^ that may contribute to VIPN pathology, and such single‐drug models may represent a significant limitation in the interpretation of the findings.

Similarly, studies that tested the effects of NLRP3 inhibition for the treatment of chemotherapy‐induced neuropathy failed to assess these effects in tumor‐bearing animals.^9^, ^14^, ^15^, ^16^ NLRP3 inflammasome signaling plays an important role in tumor progression and the response of the tumor to chemotherapy treatment. Specifically, overexpression of *NLRP3* in some types of blood cancers, including ALL and acute myeloid leukemia (AML), is associated with poor prognosis (reviewed in Urwanisch et al.^17^, ^18^). Accordingly, inhibition of NLRP3 may not only protect against the development of chemotherapy‐induced adverse effects but also have a positive impact on therapy outcomes.

To address some of these gaps, this study aims to further evaluate the impact of NLRP3 inhibition on single‐agent and combination chemotherapy‐induced adverse effects, chemotherapy efficacy, and cancer progression in NSG mice engrafted with pediatric ALL patient‐derived xenograft (PDX).

## METHODS

### Animals

All experiments were performed with female NOD/SCID/interleukin‐2 receptor *γ*‐negative (NOD.Cg‐PrkdcscidIL2rgtm1Wjl/SzJAusb, NSG) mice aged 7–9 weeks, noting that no significant differences in vincristine‐induced adverse effects were observed between sexes previously.^9^ Animals were housed in groups of three per cage under 12‐h light‐dark cycles with ad libitum access to rodent chow and water and were acclimatized to experiments as described previously.^12^ All experiments were performed by a blinded observer unaware of treatment allocations, with animals randomized to treatment groups. Appropriate sample sizes were determined by power calculation as a minimum of *n* = 4 based on an 80% power to detect an expected 60% difference relative to baseline readings with a 20% inter‐individual variability between animals and a statistical significance criterion of *p* < 0.05.

### Animal monitoring

All animals were monitored for adverse effects, specifically for weight loss and general well‐being, throughout the study and were scored for the following measures: facial grimace, locomotion, behavior, appearance, and weight (relative to weight recorded at the start of the experiment), as described in Supporting Information S1: Table 1.

### mRNA expression analysis

*NLRP3*, *CASP1*, and *IL‐1β* mRNA expression were assessed in 90 pediatric ALL PDXs by RNA sequencing (RNA‐seq) of total RNA, as described previously.^19^ All PDX models were validated before molecular characterization, as detailed previously.^20^ For the comparison of pediatric PDXs from different cancers, RNA‐seq data published in the public domain at PedcBioPortal (http://pedcbioportal.org) were used. mRNA expression in ALL patient samples was sourced from St. Jude Cloud Genomics Platform (https://platform.stjude.cloud). mRNA expression in healthy tissues was sourced from the Genotype‐Tissue Expression (GTEx) portal (version 8; https://www.gtexportal.org/home/). Read counts or fragments per kilobase of transcript per million mapped read values were converted to transcripts per million (TPM) and log2 transformed after adding a pseudo‐count of 1 (log2[TPM + 1]).

### Blood collection for whole blood analysis

Blood was collected via the lateral tail vein for complete blood count analysis performed using the BC5000 Hematology Analyzer (Mindray Bio‐Medical Electronics Co., China) in whole blood mode (23 parameters, including red blood cell count, white blood cell count, lymphocytes number, monocytes number, neutrophils number, eosinophils number, basophils number, and platelet count).

### Generation of PDX and in vivo efficacy studies

Pediatric ALL PDX model was established from primary patient samples using NOD/SCID/interleukin‐2 receptor *γ*‐negative (NOD.Cg‐*Prkdc* ^*scid*^IL2rg^*tm1Wjl*^/SzJAusb, NSG) or NOD/SCID mice, as described previously.^21^, ^22^ All cell sources used in these experiments were greater than the secondary passage. *In vivo* efficacy studies were performed as described in the Supporting Information.

### Mechanical paw withdrawal threshold (PWT) measurements

Mechanical PWTs were assessed using an electronic von Frey apparatus (MouseMet; Topcat Metrology, UK), as described previously.^12^ Before testing, animals were acclimatized (30 min) to the MouseMet test enclosures. To determine the PWT, a soft‐tipped von Frey filament was placed against the plantar surface of the hind paw, and pressure was increased linearly by rotating the handle of the device. The force required to elicit paw withdrawal was automatically determined by the TopCat software and recorded as the PWT (g). The average of three measurements per mouse, at least 10 min apart, was computed as one biological replicate.

### Motor performance assessment

Gross motor performance was assessed using the Parallel Rod Floor Test (Stoelting Co., USA) and analyzed using ANY‐Maze software (version 4.70). Animals were placed in the Parallel Rod Floor Test apparatus and allowed to acclimatize for 60 s before starting the experiment. The distance traveled (m) and the number of foot slips were recorded over 2 min using the ANY‐Maze software. The ataxia index was calculated by dividing the number of foot slips by the distance traveled (m).

### Grip‐strength meter

Deficits in the forelimb or hindlimb grip strength were assessed using the grip‐strength meter (Stoelting Co., USA), as described previously.^23^ The highest value from five independent measurements of each animal was recorded as the grip strength.

### Data and statistical analyses

Data and statistical analyses were performed using GraphPad PRISM version 10.0.00. Unless otherwise specified, statistical significance was defined as an adjusted *p* value of <0.05 and was calculated using an Ordinary One‐way ANOVA with Tukey's multiple comparison test for survival, and repeated measures two‐way ANOVA with Šídák's multiple comparisons or multiple unpaired *t*‐tests for behavioral data. All data are shown as mean ± SEM; *n* ≥ 6 for all groups, and all experimental groups were compared with the control group or baseline as indicated.

## RESULTS

### MCC950 prevents vincristine‐induced adverse effects in NSG mice

**Figure 1:**
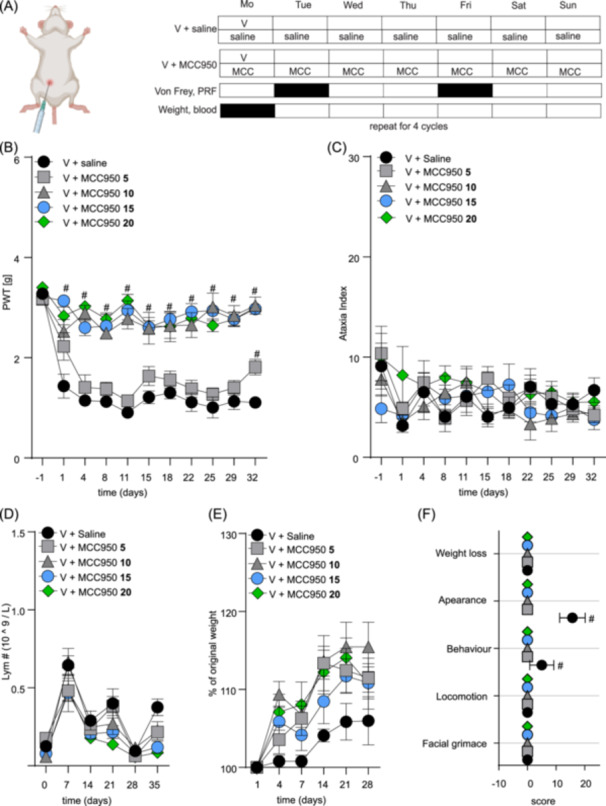
**MCC950 dose‐finding study for the prevention of mechanical allodynia, weight loss, and altered well‐being of mice treated with vincristine**. **(A)** Injection and experimental schedule of vincristine‐induced adverse effects model. Vincristine (V, 0.5 mg/kg, i.p.) was injected once a week for 4 weeks. MCC950 (5–20 mg/kg, i.p.) or saline (blue) were injected 1 h before the first vincristine administration and then daily for 4 weeks. Von Frey and parallel rod floor (PRF) apparatus assays were performed twice a week, and weight measurements and blood analyses were performed once a week as indicated in the schedule for 4 weeks. Animals were observed for an additional week (week 5). **(B)** Vincristine‐induced mechanical allodynia, measured as the decline of the mechanical paw withdrawal threshold (PWT, g) is alleviated by daily co‐administration of MCC950 (10–20 mg/kg, i.p.). **(C–E)** MCC950 had no effect on ataxia index **(C)**, lymphocyte counts **(D)**, and weight **(E)**. **(F)** Animals treated with vincristine together with increasing doses of MCC950 have lower cumulative adverse effects cores compared to animals treated with vincristine only. Statistical significance was determined by repeated measures two‐way ANOVA with Sidak's multiple comparisons test **(A–E)** or one‐way ANOVA **(F)**; data are shown as mean ± SEM. *n* ≥ 6/group. #, *p* < 0.05.

First, we examined the safety and efficacy of various doses of MCC950 (5–20 mg/kg, i.p.; 1 h before each vincristine injection and then daily for 4 weeks) in a model of vincristine‐induced adverse effects in NSG mice based on once‐weekly injection of vincristine (0.5 mg/kg, i.p.) for 4 weeks (see Figure 1A for the treatment and assessment schedule). As anticipated, higher doses of MCC950 (10, 15, and 20 mg/kg, i.p.) effectively prevented vincristine‐induced mechanical allodynia (Figure 1B) (all groups compared to V + saline group; #, *p* < 0.05) with an estimated ED_50_ of 12.0 mg/kg (90% CI 7.4–21.0). Importantly, the addition of MCC950 to vincristine did not impact motor performance, as evidenced by an unchanged ataxia index in the parallel rod floor test (Figure 1C) and did not affect lymphocyte counts (or any other blood counts, data not shown) in plasma (Figure 1D). Interestingly, treatment with all doses of MCC950 had a small, non‐significant positive effect on weight gain (Figure 1E) and an overall significant positive effect on the well‐being of the animals (Figure 1F). The administration of 5, 10, 15, or 20 mg/kg MCC950 alone did not elicit any changes in the above‐assessed parameters (data not shown).

### MCC950 co‐administration may enable vincristine therapy continuation

Vincristine‐induced adverse effects can often lead to vincristine dose reduction or discontinuation of chemotherapy. As co‐treatment with MCC950 protects from vincristine‐induced adverse effects (Figure 1), this could permit the safe continuation of vincristine‐based chemotherapy, which in turn could have beneficial effects on treatment outcomes. Therefore, we first performed a dose escalation study of vincristine (0.1–1.25 mg/kg, i.p.) during daily co‐administration of MCC950 (15 mg/kg, i.p., dose based on ED_50_ estimated in Figure 1B) and assessed effects on mechanical allodynia, motor performance (ataxia index), general well‐being measures, weight gain, and blood counts.

The doses used for the escalation study were based on the vincristine doses used to treat ALL in children,^24^ ranging from 0.03 to 3.7 mg/m^2^ human equivalent dose (HED) (0.1 mg/kg = 0.008 mg/kg/0.3 mg/m^2^ HED; 0.25 mg/kg = 0.02 mg/kg/0.7 mg/m^2^ HED; 0.5 mg/kg = 0.04 mg/kg/1.5 mg/m^2^ HED; 0.75 mg/kg = 0.06 mg/kg/2.2 mg/m^2^ HED; 1 mg/kg = 0.08 mg/kg/3 mg/m^2^ HED; 1.25 mg/kg = 0.1 mg/kg/3.7 mg/m^2^ HED).^25^

**Figure 2:**
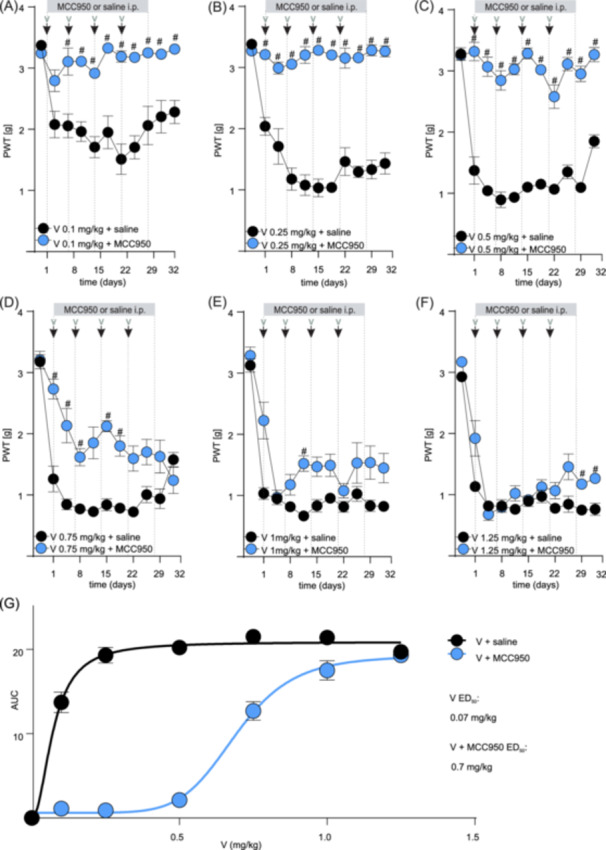
**MCC950 may allow for vincristine therapy continuation by alleviating vincristine‐induced sensory neuropathy in NSG mice. (A–C)** Mechanical allodynia induced by the treatment with lower doses of vincristine (0.1–0.5 mg/kg, i.p., black dots) is attenuated by daily co‐treatment with MCC950 (15 mg/kg, i.p., blue dots). **(D–F)** Mechanical allodynia induced by treatment with higher doses of vincristine (0.75–1.25 mg/kg, i.p., black dots) is partially attenuated by daily co‐treatment with MCC950 (15 mg/kg, i.p., blue dots). The gray bar indicates the time frame of daily MCC950 (or saline) administration. The black arrows indicate the time points of vincristine administration. **(G)** The co‐administration of MCC950 allows for the continuation of the vincristine dose by alleviating sensory neuropathy symptoms. The estimated ED_50_ of mechanical allodynia caused by vincristine used in combination with MCC950 is estimated 10× higher (0.7 mg/kg, 90% CI 0.6–0.7) compared to the ED_50_ of mechanical allodynia caused by vincristine alone (0.07 mg/kg, 90% CI 0.06–0.09). The ED_50_ was calculated using the area under the curve of the experimental values following the various doses of vincristine **(A–F)** with or without the addition of MCC950. Statistical significance was determined using repeated measures two‐way ANOVA with Sidak's multiple comparisons test. All data are shown as mean ± SEM; *n* = 6 for all groups. #, *p* < 0.05. PWT, paw withdrawal threshold.

Daily co‐administration of MCC950 (15 mg/kg, i.p.) completely prevented the decline of mechanical paw withdrawal thresholds (a measure of mechanical allodynia) in groups treated with lower doses of vincristine (0.1–0.5 mg/kg, a significant difference: V 0.1 mg/kg on days 4–32; V 0.25 mg/kg on days 1 and 8–32; V 0.5 mg/kg on days 1–32; #, *p *< 0.05, compared to V + saline‐treated control group (Figure 2A–C and Supporting Information S1: Table 2). A partially protective effect of MCC950 was also evident at 0.75 mg/kg, with limited efficacy at higher (1.0 and 1.25 mg/kg) doses of vincristine (Figure 2D–F and Supporting Information S1: Table 3). Overall, co‐treatment with MCC950 caused a significant rightward shift in the mechanical allodynia dose‐response of vincristine, with the ED_50_ in the presence of MCC950 being approximately 10‐fold higher (0.7 mg/kg, 90% CI 0.6–0.7) compared to the ED_50_ of mechanical allodynia caused by vincristine alone (0.07 mg/kg, 90% CI 0.06–0.09) (Figure 2G).

**Figure 3:**
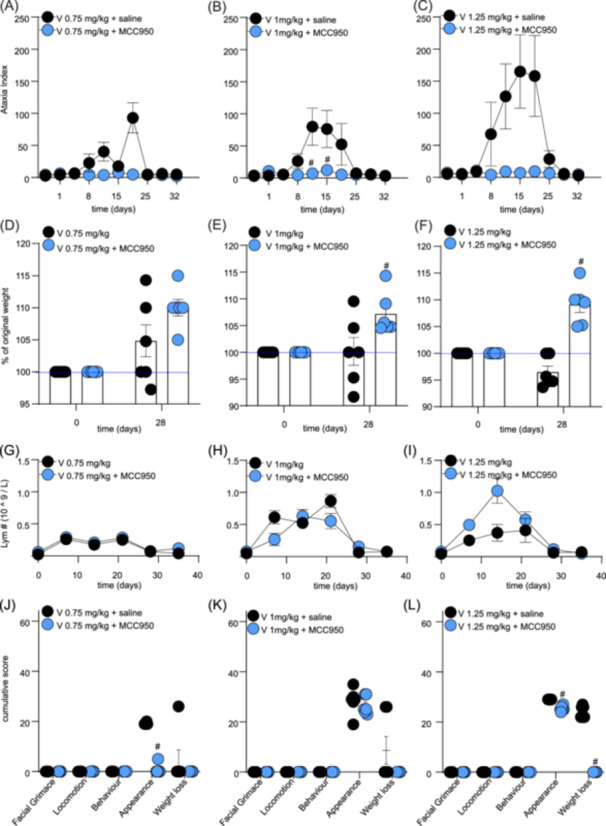
**MCC950 co‐administration alleviates adverse effects induced by high doses of vincristine. (A–C)** Motor impairment, measured as an increase in ataxia index using the Parallel Rod Floor apparatus, caused by higher doses of vincristine (0.7–1.25 mg/kg, i.p., black dots) is partially alleviated by co‐administration of MCC950 (15 mg/kg, i.p., blue dots. **(D–F)** The increase in weight over time of groups treated with higher doses of vincristine (0.75–1.25 mg/kg, i.p.) and co‐treated with MCC950 (15 mg/kg, i.p.) (blue dots) was faster compared to the groups receiving high vincristine doses only (black dots). **(G–I)** Co‐administration of MCC950 (15 mg/kg, i.p.) with high doses of vincristine (0.75–1.25 mg/kg, i.p.) (blue dots) is not significantly impacting the levels of lymphocytes in plasma compared to vincristine treatment alone (black dots). **(J–L)** Co‐administration of MCC950 (15 mg/kg, i.p.) with high doses of vincristine (0.75–1.25 mg/kg, i.p.) (blue dots) reduces cumulative scores for appearance and weight loss caused by vincristine (black dots). Statistical significance was determined using repeated measures two‐way ANOVA with Sidak's multiple comparisons test **(A–I)** or unpaired multiple *t*‐test **(J–L)**. Group comparison: V (respective dose, 0.75–1.25 mg/kg) + MCC950 compared to V (respective dose, 0.75–1.25 mg/kg) alone. All data are shown as mean ± SEM; *n* = 6 for all groups. #, *p* < 0.05.

Similarly, MCC950 also protected from vincristine‐induced effects on ataxia index and positively impacted weight gain and well‐being, albeit these effects only became significantly different from control at higher vincristine doses. Specifically, lower doses of vincristine (0.1–0.5 mg/kg) alone or in combination with MCC950 did not elicit any significant changes in ataxia index, weight gain, lymphocyte numbers, or cumulative well‐being scores (data not shown). However, a trend toward an increased ataxia index, albeit not statistically significant for most data points, emerged at higher doses of vincristine (≥1 mg/kg), which was partially prevented by co‐treatment with MCC950 (Figure 3A–C and Supporting Information S1: Table 4) (significant difference on days 11 and 15; #, *p *< 0.05, compared to V + saline‐treated control group). Furthermore, animals co‐treated with MCC950 consistently gained weight over the treatment duration, while animals receiving only vincristine lost some weight (Figure 3D–F and Supporting Information S1: Table 5) (statistical significance: V 1 mg/kg + MCC950 and V 1.25 mg/kg + MCC950 on day 28; #, *p *< 0.05, compared to V + saline‐treated control group). MCC950 did not statistically alter lymphocyte numbers (or any other blood values, data not shown) (Figure 3G–I and Supporting Information S1: Table 6). However, we observed a significant difference in cumulative scores for appearance (V 0.75 mg/kg + MCC950 and V 1.25 mg/kg + MCC950) and weight loss (V 1.25 mg/kg + MCC950) (#, *p *< 0.05, compared to V + saline‐treated control group) (Figure 3J–L and Supporting Information S1: Table 7 **)**.

### Expression of NLRP3, CASP1, and IL‐1β across various types of pediatric blood cancers

The next step was to identify the most suitable PDX model, allowing us to test the effects of MCC950 on cancer progression and chemotherapy efficiency. Although the aforementioned data suggest that inhibition of NLRP3 using the small molecule NLRP3 inhibitor MCC950 is a safe and effective treatment of VIPN in rodents, the effects of NLRP3 inhibition on cancer progression and chemotherapy efficacy are unknown. For example, glucocorticoid‐resistant leukemia cells were found to overexpress *NLRP3* and *CASP1*, suggesting that targeting NLRP3‐signaling pathways could improve response rates, particularly in treatment‐resistant diseases.^18^ Thus, we sought to assess the expression profiles of *NLRP3*, *CASP1*, and *IL‐1β* in pediatric blood cancer samples, reasoning that the largest therapeutic impact of NLRP3 inhibition on cancer progression likely occurs in leukemia subtypes with the highest expression levels.

First, we interrogated the expression of *NLRP3*, *CASP1*, and *IL‐1β* in 90 pediatric ALL PDX samples (Pediatric Preclinical Testing Consortium [PPTC] data set) that served as a platform for choosing the most suitable PDX model to study MCC950 effects on cancer. The PTCC data set includes T‐cell ALL (T‐ALL, *n* = 19), Mixed Lineage Leukemia (MLL/KMT2A)‐rearranged ALL (MLL‐ALL, n = 10), B‐cell precursor ALL (BCP‐ALL, *n* = 33), Ph‐like ALL (Ph‐like‐ALL, n = 19), Philadelphia chromosome‐positive ALL (Ph+ ALL, *n* = 3), and Early T‐cell Precursor ALL (ETP‐ALL, *n* = 6), and it is publicly available at PedcBioPortal database (http://pedcbioportal.org).^19^

**Figure 4:**
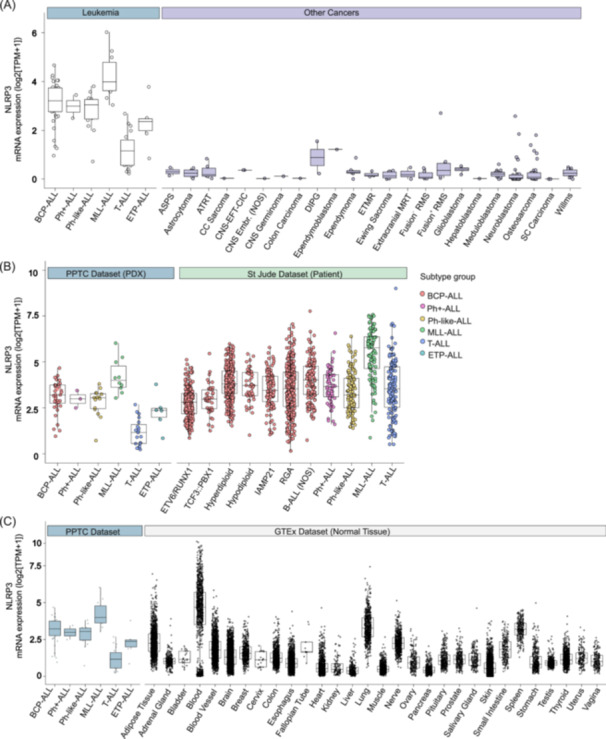
**Increased expression of NLRP3 in pediatric blood cancer types relative to other cancer types and healthy tissue. (A)** Leukemia types overexpress *NLRP3* compared to other cancer types. **(B)** The *NLRP3* expression levels of the PPTC PDX data set are similar to the *NLRP3* expression levels found in St. Jude's data set. **(C)** Comparison of the *NLRP3* expression levels in the PPTC data set to normal tissue *NLRP3* expression (GTEx data set). ASPS, alveolar soft part sarcoma; ATRT, atypical teratoid rhabdoid tumor; BCP‐ALL, B‐cell precursor ALL; CNS‐EFT‐CIC, CNS Ewing sarcoma family tumor with CIC Alteration; CNS Embr. NOS, CNS embryonal tumors, not otherwise specified; DIPG, diffuse intrinsic pontine glioma; ETMR, embryonal tumor with multilayered rosettes; ETP‐ALL, Early T‐cell Precursor ALL; iAMP, intrachromosomal amplification of chromosome 21; MLL‐ALL, MLL/KMT2A‐rearranged ALL; MRT, malignant rhabdoid tumors; NOS, not otherwise specified; Ph+ ALL, Philadelphia Chromosome‐positive ALL; Ph‐like‐ALL, Ph‐like ALL; RGA, recurrent genomic abnormalities, which encompasses B‐ALLs with known genomic abnormalities that do not have their own OncoTree subtype classification (https://oncotree.mskcc.org/)^26^; RMS, rhabdomyosarcoma; SC, small cell; T‐ALL, T‐cell ALL.

First, we compared the expression of *NLRP3* (and *IL‐1β* and *CASP‐1*) in our PTCC data set with other cancer types to demonstrate that blood cancers generally express more *NLRP3* (and *IL‐1β* and *CASP‐1*). Specifically, expression analysis revealed increased *NLRP3* expression in pediatric BCP‐ALL and MLL‐ALL PDX samples compared to the other five leukemia subtypes analyzed, with T‐ALL exhibiting the lowest expression level. Notably, in contrast to the other 23 cancer types in the PPTC study, pediatric leukemia emerges with significantly higher *NLRP3* expression (Figure 4A). Shifting focus to *IL‐1β*, our results revealed high expression in four of six leukemia subtypes, with T‐ALL and ETP‐ALL harboring the lowest expression. Moreover, BCP‐ALL, Ph+ ALL, Ph‐like‐ALL, and MLL‐ALL demonstrated the highest *IL‐1β* expression when compared to all cancer types, except for a singular case of clear cell sarcoma (Supporting Information S1: Figure 1A). With regards to *CASP‐1*, all leukemia subtypes exhibited high *CASP1* expression in comparison to other cancer types except alveolar soft part sarcoma (Supporting Information S1: Figure 1B).

Considering the inter‐individual variability in gene expression in patients, we compared our 90 pediatric ALL PDX samples (PPTC data set) with data sourced from the St. Jude Cloud Genomics Platform (https://platform.stjude.cloud; *n* = 1653) to demonstrate the variability or conservation in gene expression in various blood cancers. The comparison revealed consistent trends, with BCP‐ALL and MLL‐ALL bearing the highest *NLRP3* expression (Figure 4B), T‐ALL exhibiting the lowest *IL‐1β* expression and a persistent increase in *CASP1* expression across the leukemia panel (Supporting Information S1: Figure 2).

Finally, to demonstrate that blood cancers and hematopoietic cells express highly these genes, we leveraged the publicly available GTEx portal (version 8; https://www.gtexportal.org/home; *n* = 17,382). We observed that blood displayed high expression of *NLRP3*, *IL‐1β*, and *CASP1*, providing an important insight into the expression profiles of these inflammatory mediators in comparison to normal healthy tissues (Figure 4C and Supporting Information S1: Figure 3). The individual expression rankings of four ALL PDXs (MLL‐5, ALL‐3, MLL‐14, and ALL‐19) out of 90 PDXs are provided in Table 1, and patient demographics are shown in Supporting Information S1: Table 8.

**Table 1:** Four highest‐ranked PDX samples from 90 PDXs in the PPTC data set based on *NLRP3*, *CASP1*, and *IL‐1β* mRNA expression. Each PDX was ranked from 1 (highest) to 90 (lowest) based on mRNA expression.

Cancer ID	Subtype	Disease status at biopsy	*NLRP3* mRNA ranking	*CASP1* mRNA ranking	*IL‐1β* mRNA ranking
MLL‐5	MLL‐ALL	Diagnosis	1	2	37
ALL‐3	MLL‐ALL	Diagnosis	2	58	5
MLL‐14	MLL‐ALL	Diagnosis	3	34	65
ALL‐19	BCP‐ALL	Relapse	4	17	33

### Effect of MCC950 co‐administration on vincristine monotherapy efficacy and tumor progression in a pediatric ALL PDX model

We next sought to assess the effect of MCC950 on tumor progression and survival in PDX model. All four PDXs (Table 1) belong to ALL subtypes that are common in childhood and adult ALL. Specifically, ALL‐19 belongs to the group of BCP‐ALL, whereas ALL‐3, MLL‐14, and MLL‐5 belong to the mixed lineage leukemia (MLL/KMT2A)‐rearranged (MLL‐ALL) subtype.^27^, ^28^ However, MLL‐ALL types are generally aggressive, leading to extensive tissue infiltration and short survival of the animals in PDX models.^29^, ^30^ In contrast, BCP‐ALL types develop glucocorticoid resistance, which is driven by *NLRP3* overexpression.^18^, ^31^ Therefore, we chose the ALL‐19 PDX model to assess the effect of NLRP3 inhibition on tumor progression and vincristine monotherapy efficacy.

**Figure 5:**
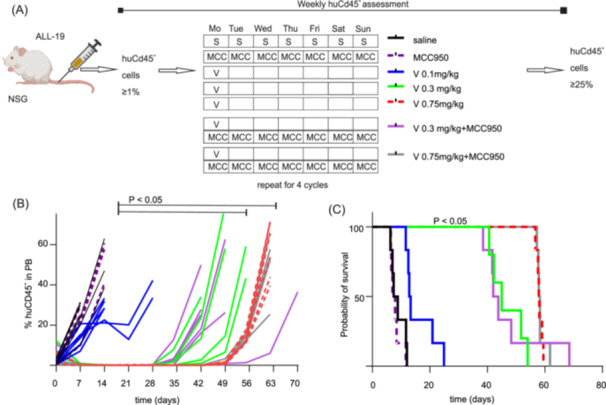
**MCC950 co‐administration has no effect on tumor progression or vincristine efficacy in ALL‐19 PDX model. (A)** ALL‐19 engraftment and injection schedule. Vincristine (0.1, 0.3, or 0.75 mg/kg, i.p.) was administered once a week for 4 weeks. MCC950 (15 mg/kg, i.p.) was administered 1 h before the first vincristine administration and then daily for 4 weeks. Human CD45^+^ lymphocytes in peripheral blood (PB) were analyzed once a week until the endpoint was reached (huCD45+ cells ≥ 25% in the PB). **(B)** Overview of ALL‐19 progression (measured as a percentage of huCD45^+^ cells in plasma) over time. Data are shown as single animal data points. **(C)** Event‐free survival (EFS): there were no significant differences in EFS of groups treated with MCC950 alone (purple dotted line) or vehicle (black line). A higher dose of vincristine 0.3 mg/kg (bright green) or 0.75 mg/kg (red) significantly (*p* < 0.05) increases the EFS compared to a low dose of vincristine 0.1 mg/kg (blue line). The addition of MCC950 to vincristine 0.3 mg/kg (violet) or 0.75 mg/kg treatment (gray) had no significant negative effect on EFS compared to vincristine 0.3 or 0.75 mg/kg treatment alone. The statistical significance of EFS was determined using ordinary one‐way ANOVA, with Tukey's multiple comparison test. Individual data are shown. *n* ≥ 6/group. #, *p* < 0.05.

Treatment with MCC950 alone (injection schedule, Figure 5A) had no effect on disease progression, as there was no significant difference in event‐free survival (median EFS; vehicle: 8.1 days; MCC950: 7.5 days) (Figure 5B). Increasing the dose of vincristine from 0.1 to 0.3 mg/kg and then to 0.75 mg/kg—the highest dose for which MCC950 was still effective at preventing mechanical allodynia (Figure 2)—significantly prolonged the EFS (V 0.1 mg/kg: median 13.1 days; V 0.3 mg/kg: median 45 days, V 0.75 mg/kg: median 58 days). Importantly, the addition of MCC950 (15 mg/kg, daily) treatment with higher doses of vincristine neither significantly affected EFS (V 0.3 mg/kg + MCC950: median 42.9 days, V 0.75 mg/kg + MCC950: median 58.1 days) (Figure 5B,C). Thus, these data suggest that MCC950 is effective at preventing vincristine‐induced side effects but does not affect treatment efficacy or disease progression.

### Effects of MCC950 co‐administration on the efficacy of combination chemotherapy

In clinical practice, vincristine is almost always administered as a part of combination chemotherapy regimens to increase the efficacy of chemotherapy and prevent the development of tumor resistance.^32^ Therefore, we next investigated the effects of MCC950 addition to the VXL (vincristine [V], dexamethasone [X], and *L*‐asparaginase [L]) regimen, a combination chemotherapy frequently used to treat ALL.^33^

We chose to test the 0.3 mg/kg dose of vincristine in this regimen, as this dose is relatively close to the vincristine doses used in the clinic (0.3 mg/kg = 0.024 mg/kg/0.8 mg/m^2^ HED) and our aforementioned data indicate that the doses between 0.25 and 0.5 mg/kg caused mild sensory neuropathy that was fully alleviated by 15 mg/kg MCC950 treatment compared to higher doses of vincristine (0.75−1.25 mg/kg), which were only partially alleviated (Figure 2). Additionally, the 0.3 mg/kg vincristine monotherapy significantly increased the EFS compared to saline‐treated group (Figure 5).

**Figure 6:**
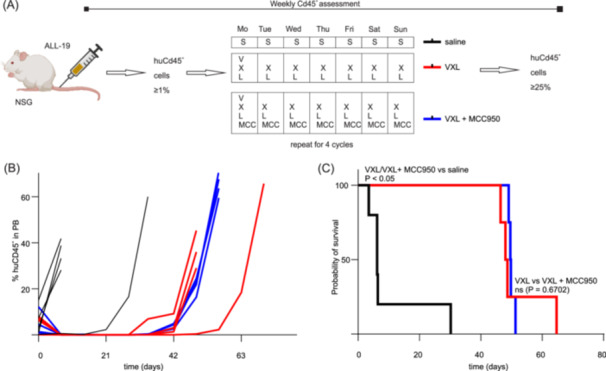
**MCC950 co‐administration has no effect on VXL efficacy and tumor progression in the ALL‐19 PDX model. (A)** ALL‐19 engraftment and injection schedule. **(B, C)** Treatment with vincristine (V), dexamethasone (X), and L‐asparaginase (L) (VXL, red lines) alone or in combination with MCC950 (VXL + MCC950, blue lines) significantly (*p* < 0.05) increase the event‐free survival (EFS) compared to saline (black lines). The addition of MCC950 to VXL (VXL + MCC950, blue lines) has no significant impact on the EFS compared to VXL alone (*p* = 0.6702) (VXL, red lines). Statistical significance of EFS was determined using ordinary one‐way ANOVA, with Tukey's multiple comparison test. Individual data are shown. *n* ≥ 6/group.

We first investigated the effects of MCC950 on tumor progression and survival in the ALL‐19 PDX model treated with the VXL combination regimen (V: 0.3 mg/kg; X: 5 mg/kg; L: 1250 KU/kg) injection schedule (Figure 6A).

The EFS of mice treated with saline was only 6.2 days. Treatment of ALL‐19‐engrafted mice with the VXL combination regimen extended the EFS to 49.7 days, and the addition of MCC950 to this regimen resulted in an EFS of 48.7 days (Figure 6B,C) with no significant difference between these two groups (*p* = 0.6702).

**Figure 7:**
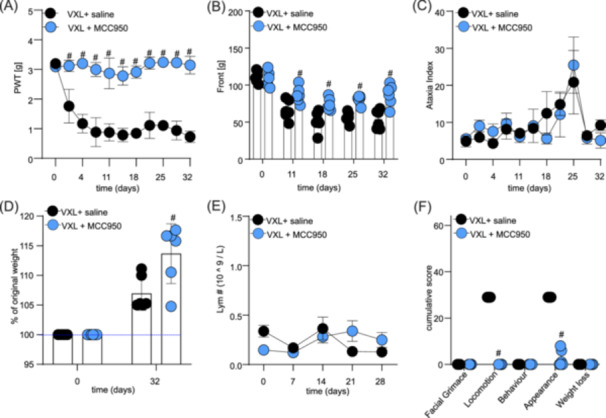
**MCC950 co‐administration prevents adverse effects induced by combination treatment with vincristine, L‐asparaginase, and dexamethasone (VXL). (A)** The combination of vincristine (0.3 mg/kg, i.p.), dexamethasone (5 mg/kg, i.p.), L‐asparaginase (1250 KU/kg, i.p.) (VXL, black dots) causes a significant decrease of paw withdrawal thresholds (PWT) in NSG mice, which was prevented by co‐treatment with MCC950 (15 mg/kg, i.p.) (VXL + MCC950, blue dots). **(B)** MCC950 co‐administration (blue dots) prevented VXL‐induced decrease in grip strength (Black dots). **(C)** Motor impairment, measured as an increase in ataxia index, caused by VXL (black dots) was unaffected by MCC950 treatment. **(D)** Significant differences in weight gain between groups treated with VXL alone (black dots) or co‐treated with MCC950 (blue dots). **(E)** Co‐administration of MCC950 with VXL (blue dots) did not significantly impact the levels of lymphocytes in plasma compared to VXL treatment alone (black dots). **(F)** Co‐administration of MCC950 with VXL (blue dots) significantly reduced cumulative adverse effects scores for locomotion and appearance caused by VXL (black dots). The statistical significance was determined using repeated measures two‐way ANOVA with Sidak's multiple comparisons test **(A–E)** or unpaired multiple *t*‐tests **(F)**. All data are shown as mean ± SEM; *n* = 6 for all groups. #, *p* < 0.05.

Additionally, the VXL regimen caused a pronounced, long‐lasting mechanical allodynia that was prevented in mice co‐treated daily with MCC950 (a significant difference on days 1–32; #, *p *< 0.05, compared to VXL + saline‐treated control group) (Figure 7A and Supporting Information S1: Table 9).

Similarly, the VXL regimen caused a decrease in forelimb grip strength measured using the grip strength meter, which was significantly prevented by daily co‐administration of MCC950 (a significant difference on days 11–32; #, *p *< 0.05, compared to VLX + saline‐treated control group) (Figure 7B and Supporting Information S1: Table 10). We also observed a significant decline in hindlimb grip strength following VXL administration, which was significantly prevented by daily co‐administration of MCC950 (data not shown) (a significant difference on days 11–25; #, *p* < 0.05, compared to VLX + saline‐treated control group).

Interestingly, VXL alone or in combination with MCC950 had no significant impact on the ataxia index (Figure 7C and Supporting Information S1: Table 11). We only observed a non‐significant increase in the ataxia index on day 25 for both groups, when compared to baseline. These data suggest that MCC950 may not have any positive effects on ataxia index increase following VXL administration.

Weight was significantly higher on day 32 in the group receiving VXL + MCC950 compared to VXL alone (VXL: 106.94 ± 2.68% of original weight vs VXL + MCC950: 113.68 ± 2.83% of original weight; #, *p *< 0.05) (Figure 7D). Additionally, there was no significant difference in whole blood lymphocyte numbers (or any other blood values, data not shown) following VXL administration (Figure 7E and Supporting Information S1: Table 12).

However, the administration of the VXL regimen caused negative changes in the overall well‐being of the animals, which were significantly prevented in the group co‐treated with MCC950 (a significant difference in appearance and locomotion score; #, *p *< 0.05, compared to VXL + saline‐treated control group) (Figure 7F and Supporting Information S1: Table 13).

Thus, these data suggest that MCC950 is effective at preventing VXL combination‐induced adverse effects but does not affect treatment efficacy or disease progression.

## DISCUSSION

In this study, we demonstrate that daily administration of 15 mg/kg MCC950 in NSG mice receiving either vincristine mono‐ or combination chemotherapy prevents symptoms of peripheral neuropathy, including mechanical hypersensitivity and a decrease in grip strength. Additionally, we demonstrate that the addition of MCC950 to vincristine mono‐ and combination chemotherapy is safe with no significant impact on ALL‐19 progression or chemotherapy efficacy.

Chemotherapy has transformed cancer outcomes and is curative in >90% of pediatric ALL cases. Although less frequent in ALL patients, compared to brain cancer patients treated with more vincristine‐intensive regimens, chemotherapy‐induced adverse effects still present an important clinical consideration for ALL treatment (reviewed in Lavoie Smith^3^ and Mora et al.^34^). The management of chemotherapy‐induced adverse effects has been hampered by a lack of understanding of the pathophysiological underlying mechanisms, as well as a lack of preclinical studies assessing treatments in tumor‐bearing animals, or with clinically relevant combination chemotherapy regimens.

Vincristine, in particular, is an important component of combination chemotherapy regimens for the treatment of childhood leukemias but causes a peripheral mixed sensory–motor neuropathy that is characterized by sensory disturbances such as pain, numbness, and tingling in the hands and feet, as well as motor impairment such as muscle weakness, difficulties in walking and running or performing fine motoric tasks.^35^

In recent years, it has become apparent that neuroinflammatory processes mediate VIPN, and specifically, that vincristine leads to activation of the NLRP3 inflammasome in monocytes, which in turn affects the release of IL‐1β that drives neuropathy symptoms, such as sensory and motor disturbances.^9^ Accordingly, inhibition of the IL‐1 receptor using anakinra or the NLRP3 inflammasome using the small molecule NLRP3 inhibitor MCC950 prevented sensory–motor neuropathy following vincristine monotherapy in mice.^9^ However, in this study, we utilized vincristine monotherapy and failed to test the effects of MCC950 on tumor progression and chemotherapy efficacy, limiting the interpretation of the findings. Therefore, the current study aimed to address these gaps and tested the efficacy and safety of MCC950 for VIPN in tumor‐bearing mice.

Interestingly, progression and poor prognosis of various leukemia types, such as high‐risk ALL, chronic myeloid leukemia, and AML, are associated with increased levels of *IL‐1β* and overexpression of *NLRP3* inflammasome (reviewed in Urwanisch et al.^17^), and overexpression of *NLRP3* and *CASP1* additionally drives glucocorticoid receptor resistance in ALL cells.^18^ Indeed, our analysis of *NLRP3* (and *CASP1* and *IL‐1β*) expression in 90 pediatric ALL PDX samples (PPTC data set)^19^ using the PedcBioPortal database confirmed higher *NLRP3* mRNA expression in ALL compared to other cancer types. Although it is not clear to what extent the RNA expression levels of the *NLRP3* gene represent the functionally active NLRP3 inflammasome, it is generally accepted that induction of *NLRP3* expression is necessary for the activation of the NLRP3 and the consequent IL‐1β release.^36^

These observations suggest that targeting NLRP3 signaling in ALL patients treated with vincristine may not only alleviate the adverse effects of chemotherapy but may additionally have positive impacts on cancer therapy. Specifically, as the success of cancer treatment using chemotherapy is dose‐dependent,^37^ minimizing dose‐dependent adverse effects could permit clinicians to treat patients who suffer higher grade vinca‐associated neurotoxicity with the recommended dose of vincristine rather than reducing the dose or omitting vincristine, as is currently practice for the treatment of chemotherapy‐induced neuropathies.

Several small‐molecule NLRP3 inflammasome inhibitors are currently in clinical studies for the treatment of neuro‐inflammatory conditions.^38^, ^39^ Thus, NLRP3 presents an attractive target for the development of effective VIPN treatment strategies. Therefore, we sought to assess the *in vivo* effects of MCC950, a specific small molecule NLRP3 inhibitor^40^ for the prevention of VIPN in NSG mice. Protection from VIPN extended to both motor and sensory symptoms in animals treated with vincristine monotherapy (Figures 2 and 3) and the VXL combination regimen, with MCC950 treatment leading to improvements in mechanical allodynia, front and hind paw grip strength, and overall animal well‐being (Figure 7). To our knowledge, this is the first time that sensory and motor neuropathy symptoms have been reported from a murine model of combination chemotherapy with the VXL regimen.

Paugh et al. demonstrated that glucocorticoid resistance in ALL cells isolated from patients is driven by increased expression of *NLRP3* and *CASP1* genes and the consequent cleavage of the glucocorticoid receptors by CASP1.^18^ These findings suggest that NLRP3 inhibition using MCC950 should improve the response of glucocorticoid‐resistant ALL‐19 to treatment. However, our results suggest that such effects are unlikely in our animal model, given that we assessed EFS following co‐treatment with MCC950 in the PDX model with the highest expression levels of *NLRP3* and demonstrated that the addition of MCC950 to the VXL regimen did not impact chemotherapeutic efficacy or EFS (Figure 6). Our study utilized ALL‐19 PDX in NSG mice *in vivo*, whereas Paugh et al. tested patient‐derived cell lines *in vitro*. It is also unclear whether ALL‐19 was included in the study performed by Paugh et al. possibly, explaining the differences between these findings. Therefore, further studies directly addressing whether glucocorticoid resistance is improved by NLRP3 inhibition *in vivo* are required.

More broadly, our results may have implications for other classes of chemotherapeutic agents, as similar neuroinflammatory mechanisms contribute to the development of adverse effects following the treatment with paclitaxel, doxorubicin, and epothilones.^9^, ^41^, ^42^ Our studies were performed in rodents, specifically NSG mice, and it is currently unclear to what extent these findings can be translated to patients. Specifically, in this study, chemotherapy drugs were administered intraperitoneally, and the intra‐ and interspecies differences in chemotherapy kinetics may impact the future translation of our findings.^43^ Additionally, the relatively short axon length in rodents compared to humans may limit the comparison with the clinical scenario. Nonetheless, rodent models are still the standard of preclinical testing, and these results provide proof of principle that inhibition of NLRP3 reduces adverse effects of vincristine mono‐ and combination chemotherapy in NSG mice.

Although the IL‐1 receptor antagonist anakinra was also efficacious in preclinical studies assessing effects on vincristine‐induced neuropathy, inhibition of the IL‐1 receptor is known to detrimentally affect immune function. In contrast, inhibition of the NLRP3 inflammasome offers a targeted approach that is expected to have a minor impact on the patient's immune system, as IL‐1 is also regulated by other inflammasomes.^44^ Although MCC950 was reported to cause hepatotoxicity in a Phase II clinical trial for the treatment of rheumatoid arthritis,^45^ Selnofast, a less toxic derivate of the MCC950, is currently in Phase 1 clinical trials by Roche (e.g., trial ID: GC43343), possibly offering a future targeted approach for the treatment of chemotherapy‐induced adverse effects.

## AUTHOR CONTRIBUTIONS

Hana Starobova, Hannah McCalmont, Richard B. Lock, and Irina Vetter participated in the research design. Hana Starobova, Hannah McCalmont, Nicolette Tay, and Svetlana Shatunova conducted the experiments. Hana Starobova, Hannah McCalmont, and Christopher M. Smith carried out the data analysis. Avril Robertson synthesized MCC950. Ingrid Winkler provided laboratory resources and laboratory space. Hana Starobova, Hannah McCalmont, Christopher M. Smith, Richard B. Lock, and Irina Vetter wrote the manuscript.

## CONFLICT OF INTEREST STATEMENT

The authors declare no conflicts of interest.

## FUNDING

Starobova was supported by the Children's Hospital Foundation Fellowship, Lock was supported by a Fellowship from the Australian National Health and Medical Research Council, and Vetter was supported by NHMRC Investigator grant 2017086. This work was supported by the Kids' Cancer Project.

## Supporting information

Supporting information.

## Data Availability

The data sets generated during and/or analyzed during the current study are available from the corresponding author upon reasonable request.
