# Supplementary material for: Inhibition of the NLRP3 inflammasome using MCC950 reduces vincristine‐induced adverse effects in an acute lymphoblastic leukemia patient‐derived xenograft model
Source: Hemasphere. 2025 Mar 18;9(3):e70092. doi: 10.1002/hem3.70092 (PMC11915122; doi:10.1002/hem3.70092)
Supplement: Supplementary file 1 — Supporting information. [file HEM3-9-e70092-s001.pdf]

Supplementary material

Inhibition of the NLRP3 inflammasome using MCC950 reduces vincristine-induced adverse effects in an acute lymphoblastic leukemia patient-derived xenograft model

Hana Starobova [1*], Hannah McCalmont [2], Svetlana Shatunova [3], Nicolette Tay [1], Christopher M. Smith [2], Avril Robertson [1,4], Ingrid Winkler [3], Richard B. Lock [2] and Irina Vetter [1,5]

*Corresponding Author

Affiliations:

[1] Institute for Molecular Bioscience, The University of Queensland, St Lucia, QLD, Australia.

[2] Children's Cancer Institute, Lowy Cancer Research Centre, School of Clinical Medicine, UNSW Medicine & Health, UNSW Centre for Childhood Cancer Research, UNSW Sydney, Sydney, NSW, Australia.

[3] Mater Research Institute-The University of Queensland, South Brisbane, QLD, Australia.

[4] School of Chemistry and Molecular Biosciences, The University of Queensland, St Lucia, QLD, Australia.

[5] The School of Pharmacy, The University of Queensland, Woolloongabba, QLD, Australia.

1.1. Supplementary methods:

Ethical approvals

All experiments were performed in accordance with the 2012 Animal Care and Protection Regulation Qld; the 2013 Australian Code of Practice for the Care and Use of Animals for Scientific Purposes (8th edition); and the International Association for the Study of Pain Guidelines for the Use of Animals in Research. Patient-derived xenografts (PDXs) were established and used under the approval of the University of New South Wales Animal Care and Ethics Committee. For behavioral studies, ethical approval was obtained from the University of Queensland.

Animal Monitoring

All animals were monitored for adverse events, specifically for weight loss and general well-being, throughout the study and were scored for the following measures: facial grimace, locomotion, behavior, appearance and weight (relative to weight recorded at the start of the experiment). Scores of 3 in any category were defined as requiring immediate euthanasia (detailed in **Supplementary Table 1**). The maximum cumulative adverse events score an animal could have reached without being euthanized over 5 weeks of the experiment was 70 (max adverse event score for the day (2) x the number of days in the experiment (35)). All experiments were conducted by blinded observer unaware of the treatment, in cohorts with experimental groups or vehicle controls assessed at the same time, and only cohort-specific control animals were used for the statistical analysis of each treatment.

Supplementary Table 1: The scoring system used to assess animal wellbeing.

Criteria	Score			
	0	1 (mild)	2 (moderate	3 (severe)
facial grimace	not present	facial grimace is subtle or inconsistent	facial grimace is moderate	facial grimace is obvious
locomotion	walking normally	limping, stiffness	swollen limbs	severely restricted mobility
behaviour	normal behaviour	away from littermates	aggressive or huddled in the corner	severe distress
appearance	normal	ruffled fur	animal appears depressed, hunched, reluctant to move	animal appears severely depressed
Weight loss, relative to weight recorded at the start of the experiment	<5%	5%-9%	10%-14%	≤15%

In vivo efficacy studies:

Vincristine, MCC950 and VXL were evaluated *in vivo* against a B-cell precursor (BCP)-ALL PDX model (1, 2), ALL-19. Cells were inoculated via tail vein injection (3 x 10^6 cells per mouse, n=6/group) into NSG mice (Australian BioResources, Moss Vale NSW, Australia) in a volume of 100 µL calcium- and magnesium-free phosphate-buffered saline (PBS). Peripheral blood (PB) sampling via tail vein bleeds was performed weekly to monitor leukemia engraftment and assess drug response by enumerating the proportion of human ($huCD45^+$) versus mouse ($muCD45^+$) cells in the PB. Samples were stained with anti-CD45 antibodies, fluorescein isothiocyanate (FITC)-conjugated antimurine (muCD45) and allophycocyanin-conjugated antihuman (huCD45) (BD Biosciences) and assessed using the FACSCanto (BD Biosciences) flow cytometer. Drug treatments began once the median percentage of $huCD45^+$ cells reached ≥1% for each treatment group and the experimental endpoint pre-determined as the $\%huCD45^+$ reaching 25% in the PB, indicative of systemic disease. Individual

mouse event-free survival (EFS) was calculated as the number of days from Day 0 (treatment initiation) until %huCD45^{+} reached 25% by interpolating between bleed values immediately preceding, and following, 25% huCD45^{+} and assumes a log-linear growth in leukemia progression. Vincristine (V) was administered via intraperitoneal (i.p.) injection once weekly for 4 weeks at a dose of 0.1 mg/kg, 0.3 mg/kg or 0.75 mg/kg as a single agent and a dose of 0.3 mg/kg in combination with 5 mg/kg dexamethasone (X) and 1250 KU/kg *L*-asparaginase (L) in a VXL combination treatment (XL administered via i.p. injection 5 days on, 2 days off for 4 weeks). For VXL treatments, vincristine and dexamethasone were purchased from Clifford Hallam Healthcare (NSW, Australia) and *L*-asparaginase purchased from Orphama (VIC, Australia). MCC950 (3) was diluted in phosphate buffered saline (PBS) and administered at a dose of 15 mg/kg once daily via i.p. as indicated in treatment schedules for 28 days. The dose of MCC950 used in the study was determined based on previously published experiments (4) and the experiment presented in **Figure 1**. VXL combination treatment doses against PDX models *in vivo* used in this study are based on previously published studies (5-8).

1.2. Supplementary results

Supplementary Table 2: Systemic administration of lower doses of vincristine (0.1 – 0.5 mg/kg) elicits dose-dependent mechanical allodynia in NSG mice that is significantly prevented by co-administration of MCC950. Statistical significance was determined by repeated measures two-way ANOVA with Sidak's multiple comparisons test and defined as $*p < 0.05$ compared to V + Saline control. All data are shown as mean ± SEM; n = 6 for all groups.

Day	V 0.1 mg/kg + Saline		V 0.1 mg/kg + MCC950 15 mg/kg		V 0.25 mg/kg + Saline kg + mg/kg		V 0.25 mg/kg + MCC950 15 mg/kg		V 0.5 mg/kg + Saline		V 0.5 mg/kg + MCC950 15 mg/kg	
	**Mean (g)**	**SEM (g)**	**Mean (g)**	**SEM (g)**	**Mean (g)**	**SEM (g)**	**Mean (g)**	**SEM (g)**	**Mean (g)**	**SEM (g)**	**Mean (g)**	**SEM (g)**
**-1**	3.37	0.06	3.24	0.08	3.38	0.08	3.27	0.06	3.28	0.10	3.26	0.07
**1**	2.08	0.22	2.79	0.18	2.04	0.15	*3.21	0.07	1.37	0.22	*3.32	0.15
**4**	2.06	0.19	*3.11	0.22	1.71	0.29	*2.99	0.10	1.04	0.08	*3.07	0.16
**8**	1.96	0.16	*3.11	0.06	1.18	0.18	*3.06	0.08	0.89	0.13	*2.84	0.16
**11**	1.71	0.17	*2.92	0.07	1.08	0.17	*3.21	0.14	0.93	0.03	*3.03	0.09
**15**	1.95	0.27	*3.33	0.07	1.03	0.14	*3.28	0.08	1.10	0.04	*3.29	0.09
**18**	1.51	0.25	*3.19	0.09	1.04	0.08	*3.21	0.06	1.15	0.07	*3.02	0.08
**22**	1.71	0.19	*3.18	0.06	1.46	0.23	*3.16	0.16	1.07	0.06	*2.58	0.19
**25**	2.06	0.31	*3.25	0.09	1.29	0.09	*3.16	0.04	1.35	0.11	*3.11	0.11
**29**	2.21	0.27	*3.23	0.05	1.33	0.15	*3.28	0.09	1.09	0.08	*2.95	0.13
**32**	2.28	0.19	*3.31	0.05	1.43	0.17	*3.27	0.09	1.85	0.11	*3.27	0.12

Supplementary Table 3: Systemic administration of various doses of vincristine (0.75 – 1.25 mg/kg) elicits dose-dependent mechanical allodynia in NSG mice that is partially significantly prevented by co-administration of MCC950. Statistical significance was determined by repeated measures two-way ANOVA with Sidak's multiple comparisons test and defined as $*p < 0.05$ compared to V + Saline control. All data are shown as mean ± SEM; n = 6 for all groups.

**Day**	**V 0.75 mg/kg + Saline**		**V 0.75 mg/kg + MCC950 15 mg/kg**		**V 1mg/kg + Saline**		**V 1mg/kg + MCC950 15 mg/kg**		**V 1.25 mg/kg + Saline**		**V 1.25 mg/kg + MCC950 15 mg/kg**	
	**Mean (g)**	**SEM (g)**	**Mean (g)**	**SEM (g)**	**Mean (g)**	**SEM (g)**	**Mean (g)**	**SEM (g)**	**Mean (g)**	**SEM (g)**	**Mean (g)**	**SEM (g)**
**-1**	3.18	0.11	3.21	0.14	3.13	0.11	3.29	0.14	2.93	0.04	3.17	0.07
**1**	1.26	0.21	*2.73	0.16	1.03	0.10	2.23	0.30	1.13	0.08	1.92	0.29
**4**	0.84	0.09	*2.13	0.28	0.95	0.06	0.97	0.12	0.82	0.08	0.68	0.10
**8**	0.77	0.05	*1.62	0.14	0.82	0.08	1.18	0.17	0.82	0.10	0.79	0.05
**11**	0.73	0.05	1.85	0.26	0.67	0.07	*1.52	0.12	0.76	0.08	1.02	0.12
**15**	0.84	0.10	*2.12	0.09	0.83	0.10	1.47	0.16	0.89	0.09	0.92	0.07
**18**	0.78	0.08	*1.80	0.17	0.96	0.07	1.49	0.18	0.97	0.10	1.13	0.12
**22**	0.72	0.08	1.59	0.21	0.82	0.09	1.08	0.12	0.78	0.06	1.07	0.13
**25**	1.01	0.13	1.70	0.21	1.03	0.12	1.53	0.33	0.84	0.13	1.46	0.21
**29**	0.94	0.16	1.63	0.27	0.83	0.09	1.54	0.27	0.75	0.07	*1.17	0.04
**32**	1.58	0.12	1.24	0.21	0.82	0.07	1.45	0.24	0.76	0.10	*1.27	0.08

Supplementary Table 4: Systemic administration of various doses of vincristine (0.75 – 1.25 mg/kg) elicits an increase of the ataxia index in NSG mice that is partially significantly prevented by co-administration of MCC950. Statistical significance was determined by repeated measures two-way ANOVA with Sidak's multiple comparisons test and defined as $*p < 0.05$ compared to V + Saline control. All data are shown as mean ± SEM; n = 6 for all groups.

Day	V 0.75 mg/kg + Saline		V 0.75 mg/kg + MCC950 15 mg/kg		V 1mg/kg + Saline		V 1mg/kg + MCC950 15 mg/kg		V 1.25 mg/kg + Saline		V 1.25 mg/kg + MCC950 15 mg/kg	
	**Mean**	**SEM**	**Mean**	**SEM**	**Mean**	**SEM**	**Mean**	**SEM**	**Mean**	**SEM**	**Mean**	**SEM**
**-1**	2.83	0.55	4.43	0.76	3.03	0.65	3.64	0.61	5.43	1.78	7.27	1.56
**1**	4.30	1.29	6.34	1.36	3.63	0.65	10.7	2.95	5.60	1.21	4.52	1.21
**4**	5.93	1.55	6.64	3.04	5.48	0.97	5.50	1.81	8.96	2.56	10.44	5.20
**8**	22.80	14.00	4.16	0.81	26.29	11.4	4.63	0.81	67.44	49.8	4.14	0.88
**11**	40.42	14.74	3.85	1.07	80.01	29.1	*6.89	1.38	126.25	50.7	9.23	1.74
**15**	17.73	6.84	7.75	2.81	76.29	29.0	*12.3	2.86	164.93	57.3	7.05	1.24
**18**	92.99	23.60	4.84	1.11	52.49	32.4	5.25	0.92	157.97	62.8	9.50	2.39
**22**	4.83	0.64	4.27	0.76	7.25	0.89	5.26	0.80	28.59	13.1	6.16	1.23
**25**	5.95	1.65	3.93	0.66	5.85	1.19	5.44	0.89	5.79	1.46	4.44	0.74
**29**	4.92	1.14	2.42	0.65	3.57	0.51	3.28	0.53	3.15	1.12	4.71	1.55
**32**	2.83	0.55	4.43	0.76	3.03	0.65	3.64	0.61	5.43	1.78	7.27	1.56

Supplementary Table 5: Systemic administration of various doses of vincristine (0.75 – 1.25 mg/kg) elicits a slower increase or small decrease in weight in NSG mice that is partially significantly prevented by co-administration of MCC950. Statistical significance was determined by repeated measures two-way ANOVA with Sidak's multiple comparisons test and defined as $*p < 0.05$ compared to V + Saline control. All data are shown as mean ± SEM; n = 6 for all groups.

Day	V 0.75 mg/kg + Saline		V 0.75 mg/kg + MCC950 15 mg/kg		V 1mg/kg + Saline		V 1mg/kg + MCC950 15 mg/kg		V 1.25 mg/kg + Saline		V 1.25 mg/kg + MCC950 15 mg/kg	
	**Mean (%)**	**SEM (%)**	**Mean (%)**	**SEM (%)**	**Mean (%)**	**SEM (%)**	**Mean (%)**	**SEM (%)**	**Mean (%)**	**SEM (%)**	**Mean (%)**	**SEM (%)**
0	100.0	0.0	100.0	0.0	100.0	0.0	100.0	0.0	100.0	0.0	100.0	0.0
28	104.8	2.5	110.0	1.3	100.2	2.6	*107.2	1.6	96.47	1.1	*109.1	1.5

Supplementary Table 6: Systemic administration of various doses of vincristine (0.75 – 1.25 mg/kg) has no significant effects on whole blood lymphocyte numbers in NSG mice. Statistical significance was determined by repeated measures two-way ANOVA with Sidak's multiple comparisons test and defined as $*p < 0.05$ compared to V + Saline control. All data are shown as mean ± SEM; n = 6 for all groups.

Day	V 0.75 mg/kg + Saline		V 0.75 mg/kg + MCC950 15 mg/kg		V 1mg/kg + Saline		V 1mg/kg + MCC950 15 mg/kg		V 1.25 mg/kg + Saline		V 1.25 mg/kg + MCC950 15 mg/kg	
	Mean (10^9/L)	SEM (10^9/L)	Mean (10^9/L)	SEM (10^9/L)	Mean (10^9/L)	SEM (10^9/L)	Mean (10^9/L)	SEM (10^9/L)	Mean (10^9/L)	SEM (10^9/L)	Mean (10^9/L)	SEM (10^9/L)
0	0.025	0.007	0.058	0.012	0.050	0.009	0.078	0.026	0.043	0.010	0.075	0.013
7	0.260	0.042	0.288	0.035	0.613	0.105	0.268	0.094	0.253	0.051	0.495	0.089
14	0.175	0.026	0.208	0.054	0.525	0.081	0.633	0.103	0.372	0.129	1.027	0.193
21	0.252	0.021	0.283	0.082	0.867	0.103	0.553	0.118	0.410	0.187	0.577	0.121
28	0.067	0.017	0.073	0.012	0.065	0.012	0.157	0.042	0.062	0.011	0.113	0.024
35	0.040	0.004	0.117	0.035	0.077	0.033	0.077	0.012	0.070	0.024	0.050	0.010

Supplementary Table 7: Systemic administration of various doses of vincristine (0.75 – 1.25 mg/kg) elicits an increase of cumulative scores in NSG mice that is significantly prevented by co-administration of MCC950. Statistical significance was determined by multiple unpaired t-test and defined as $*p < 0.05$ compared to V + Saline control. All data are shown as mean ± SEM; n = 6 for all groups.

Day	V 0.75 mg/kg + Saline		V 0.75 mg/kg + MCC950 15 mg/kg		V 1mg/kg + Saline		V 1mg/kg + MCC950 15 mg/kg		V 1.25 mg/kg + Saline		V 1.25 mg/kg + MCC950 15 mg/kg	
	Mean	SEM	Mean	SEM	Mean	SEM	Mean	SEM	Mean	SEM	Mean	SEM
**Facial grimace**	0.00	0.00	0.00	0.00	0.00	0.00	0.00	0.00	0.00	0.00	0.00	0.00
**Locomotion**	0.00	0.00	0.00	0.00	0.00	0.00	0.00	0.00	0.00	0.00	0.00	0.00
**Behavior**	0.00	0.00	0.00	0.00	0.00	0.00	0.00	0.00	0.00	0.00	0.00	0.00
**Appearance**	19.17	0.17	*0.83	0.83	28.33	2.12	26.50	1.45	29.00	0.00	*25.33	0.42
**Weight loss**	4.33	4.33	0.00	0.00	8.67	5.48	0.00	0.00	24.17	0.98	*0.00	0.00

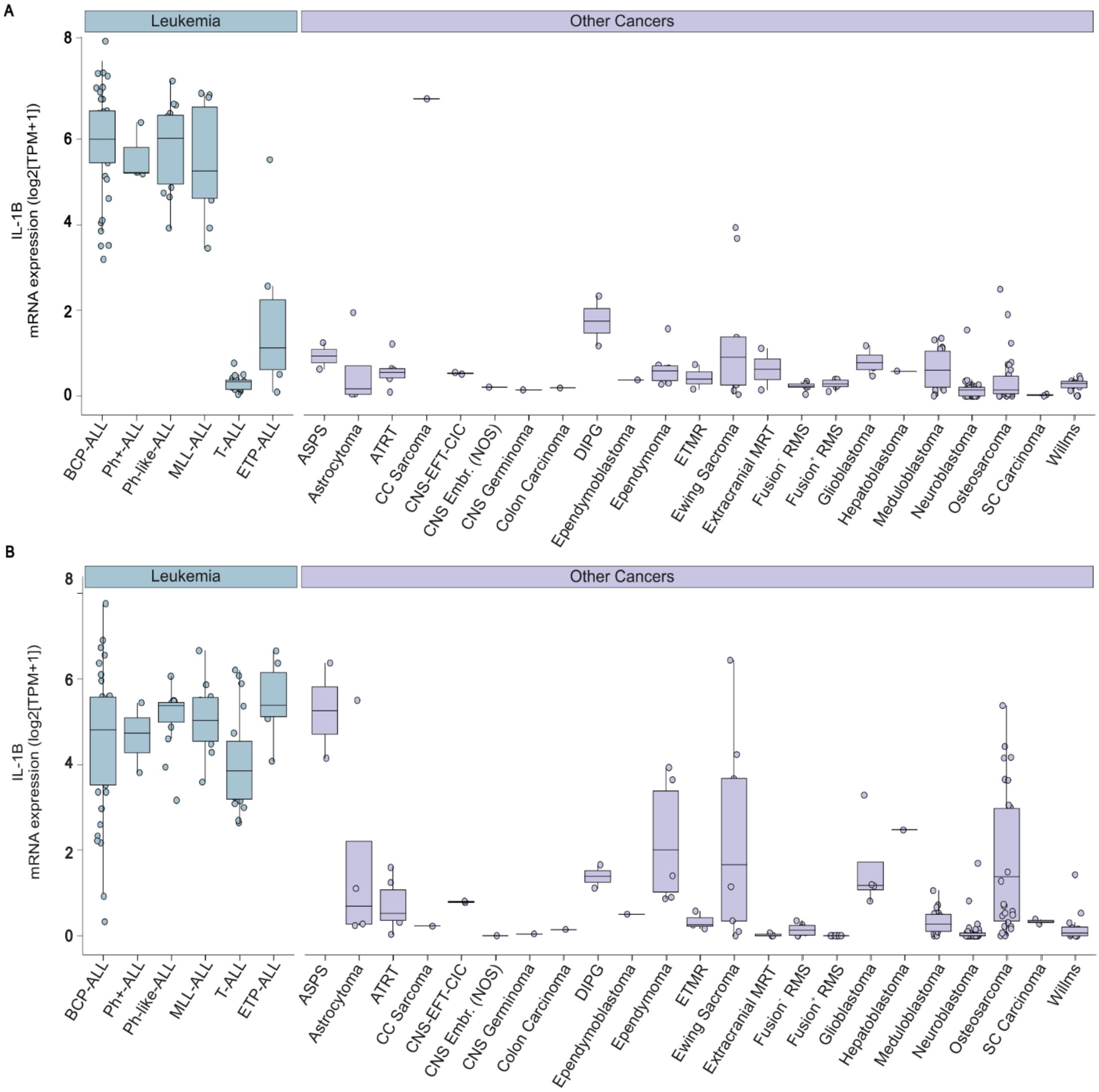

Supplementary Figure 1: *Increased expression of IL-1β (IL-1B) and caspase 1 (CASP1) in pediatric blood cancer types relative to other cancer types*. *BCP-ALL: B-cell precursor ALL, T-ALL: T-cell ALL, MLL-ALL: MLL/KMT2A-rearranged ALL, Ph+-ALL: Philadelphia Chromosome–positive ALL, Ph-like-ALL: Ph-like ALL, ETP-ALL: Early T-cell Precursor ALL, ASPS: Alveolar Soft Part Sarcoma, ATRT: Atypical Teratoid Rhabdoid Tumor, CNS-EFT-CIC: CNS Ewing Sarcoma Family Tumor with CIC Alteration, CNS Embr. NOS: CNS Embryonal Tumors, Not Otherwise Specified (NOS), DIPG: Diffuse Intrinsic Pontine Glioma, ETMR: Embryonal Tumor with Multilayered Rosettes, MRT: Malignant Rhabdoid Tumors, RMS: Rhabdomyosarcoma, SC: Small Cell.*

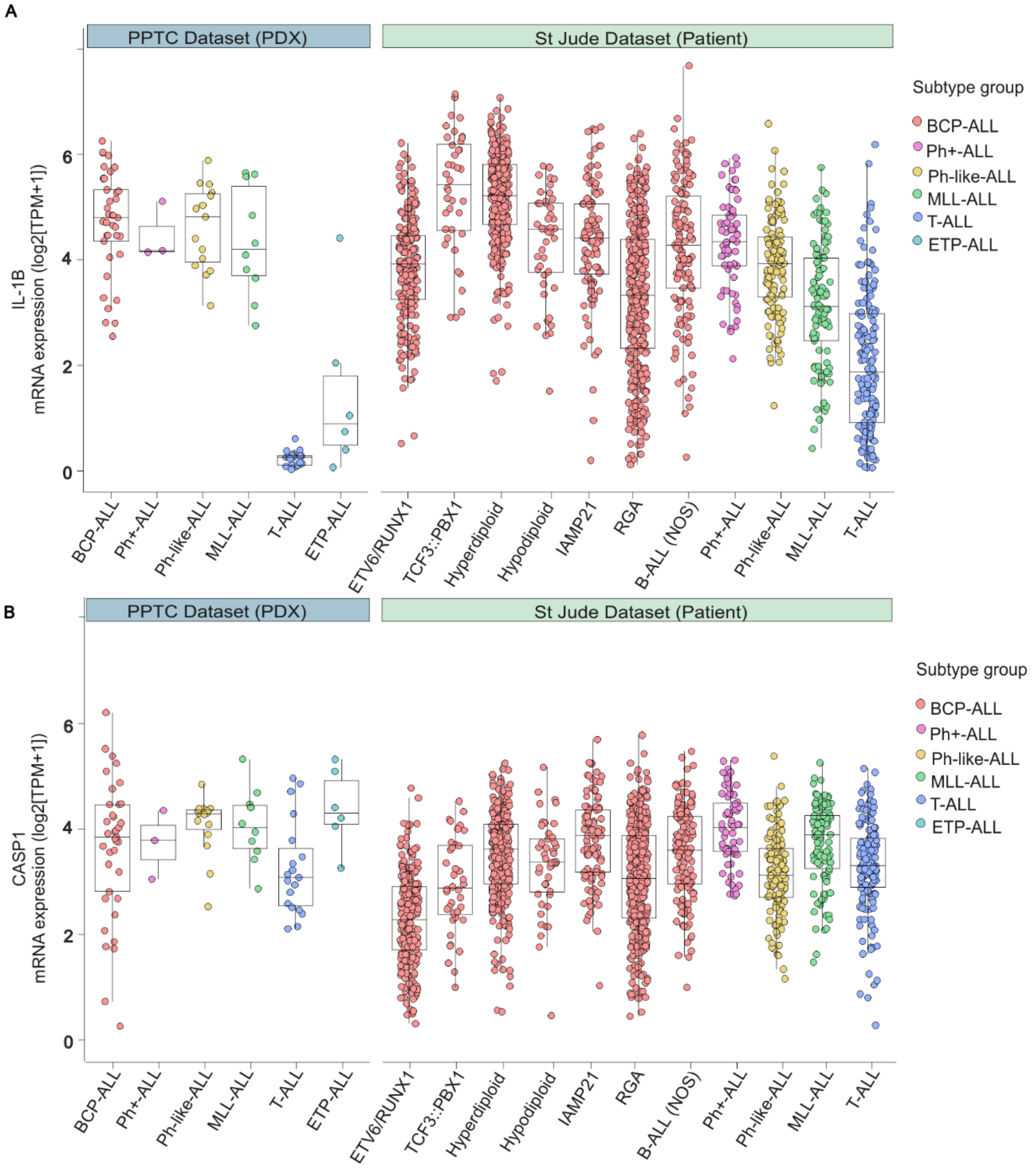

Supplementary Figure 2: ***Expression of IL-1β (IL-1B) and caspase 1 (CASP1) in Patient-Derived Xenografts of blood cancers (PPTC dataset) compared to patient samples of blood cancer (St. Judes Dataset*** *BCP-ALL: B-cell precursor ALL, T-ALL: T-cell ALL, MLL-ALL: MLL/KMT2A-rearranged ALL, Ph+-ALL: Philadelphia Chromosome–positive ALL, Ph-like-ALL: Ph-like ALL, ETP-ALL: Early T-cell Precursor ALL, iAMP: Intrachromosomal Amplification of Chromosome 21, RGA: Recurrent Genomic Abnormalities, which encompasses B-ALLs with known genomic abnormalities that do not have their own OncoTree subtype classification (*https://oncotree.mskcc.org/*), NOS: Not Otherwise Specified (NOS).*

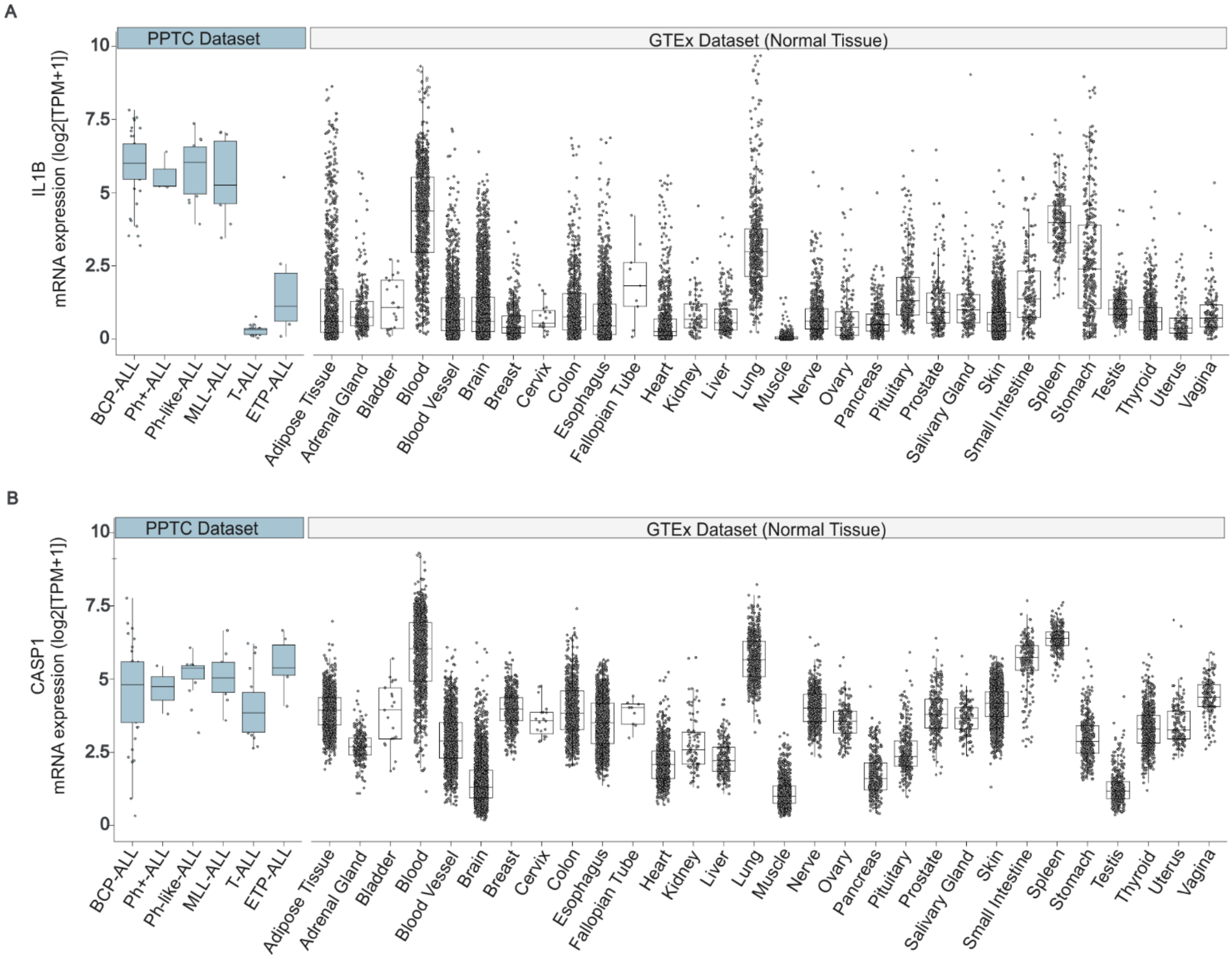

Supplementary Figure 3: ***Expression of IL-1β (IL-1B) and caspase 1 (CASP1) in Patient-Derived Xenografts of blood cancers (PPTC dataset) compared to other types of tissue (GTEx dataset, normal tissue).*** *BCP-ALL: B-cell precursor ALL, T-ALL: T-cell ALL, MLL-ALL: MLL/KMT2A-rearranged ALL, Ph+-ALL: Philadelphia Chromosome–positive ALL, Ph-like-ALL: Ph-like ALL, ETP-ALL: Early T-cell Precursor ALL.*

Supplementary Table 8: Patient demographics for MLL-5, ALL-3, MLL-14 and ALL-19 PDXs.

PDX	MLL-5	ALL-3	MLL-14	ALL-19
**Stage of disease**	Diagnosis	Diagnosis	Diagnosis	Relapse
**Sex**	Male	Female	Female	Male
**Age**	<1 y	13 y	< 1 y	16 y
**Fusion**	KMT2A:MLLT10;PAX5:ZCCHC7	KMT2A:MLLT1;PAX5:ZCCHC7	KMT2A:MLLT1	NUP214:ABL1
**Mutations (AA, VAF)**	AKT1 (R465H; 0.44); HAO2 (R336W; 0.40)	CTCF (A648T, 0.53); ALK (A17T, 0.47); ALK (S15Y, 0.44); BAZ1A (R1480H, 0.47); KMT2D (G1384D, 0.48); KMT2D (G1384S, 0.49); MGA (E2760K, 0.54)	LRIG1 (L288F, 0.46); KMT2D (P2301S, 0.51); KMT2D (R5007W, 0.46); MYH11 (L1004V, 0.52); ATR (L2076V, 0.55)	MLLT4 (P443R, 0.47); KRAS (L23R, 0.54); GNB1 (89_90insD, 0.49); FLG (S1848F, 0.56)
**Length of CR1**	Unknown	Unknown	Unknown	4 months
**Survival after relapse**	Unknown	Unknown	Unknown	7 months
**Outcome**	Unknown	Unknown	Unknown	Died of disease

Supplementary Table 9: Systemic administration of VXL combination regimen (vincristine: V, 0.3 mg/kg; dexamethasone: X, 5mg/kg, *L*-asparaginase: L, 1250 KU/kg) elicits mechanical allodynia in NSG mice that is significantly prevented by co-administration of MCC950. Statistical significance was determined by repeated measures two-way ANOVA with Sidak's multiple comparisons test and defined as $*p < 0.05$ compared to VXL + saline control. All data are shown as mean ± SEM; n = 6 for all groups.

Day	VXL + saline		VXL + MCC950 15 mg/kg	
	Mean (g)	SEM (g)	Mean (g)	SEM (g)
-1	3.19	0.05	3.09	0.07
1	1.76	0.23	*3.12	0.08
4	1.17	0.13	*3.20	0.03
8	0.88	0.20	*2.99	0.10
11	0.87	0.12	*2.87	0.21
15	0.78	0.09	*2.78	0.13
18	0.84	0.03	*2.91	0.09
22	1.11	0.18	*3.22	0.08
25	1.11	0.07	*3.24	0.06
29	0.94	0.13	*3.23	0.07
32	0.73	0.08	*3.14	0.12

Supplementary Table 10: Systemic administration of VXL combination regimen (vincristine: V, 0.3 mg/kg; dexamethasone: X, 5 mg/kg, *L*-asparaginase: L, 1250 KU/kg) elicits a significant decrease of front leg grip strength in NSG mice that is significantly prevented by co-administration of MCC950. Statistical significance was determined by repeated measures two-way ANOVA with Sidak's multiple comparisons test and defined as $*p < 0.05$ compared to VXL + saline control. All data are shown as mean ± SEM; n = 6 for all groups.

Day	VXL + saline		VXL + MCC950 15 mg/kg	
	Mean (g)	SEM (g)	Mean (g)	SEM (g)
-1	111.37	3.12	107.71	4.33
11	66.32	5.30	*86.74	4.36
18	50.95	5.34	*73.66	3.34
25	56.02	2.93	*80.53	2.37
32	53.95	5.10	*86.82	5.79

Supplementary Table 11: Systemic administration of VXL combination regimen (vincristine: V, 0.3 mg/kg; dexamethasone: X, 5 mg/kg, *L*-asparaginase: L, 1250 KU/kg) has no negative impact on the ataxia index in NSG mice. Statistical significance was determined by repeated measures two-way ANOVA with Sidak's multiple comparisons test and defined as $*p < 0.05$ compared to VXL + saline control. All data are shown as mean ± SEM; n = 6 for all groups.

Day	VXL + saline		VXL + MCC950 15 mg/kg	
	Mean	SEM	Mean	SEM
-1	4.88	1.51	5.58	1.03
1	5.94	0.98	9.03	1.58
4	4.30	1.05	7.54	2.04
8	8.15	2.69	9.72	2.80
11	7.07	1.07	6.15	1.59
15	8.42	1.35	9.06	4.52
18	12.46	5.88	5.65	1.48
22	14.86	3.01	12.13	6.09
25	20.84	8.57	25.46	7.59
29	6.43	0.89	5.80	1.68
32	9.23	1.41	5.15	2.08

Supplementary Table 12: Systemic administration of VXL combination regimen (vincristine: V, 0.3 mg/kg; dexamethasone: X, 5 mg/kg, *L*-asparaginase: L, 1250 KU/kg) has no negative impact on the peripheral blood lymphocytes numbers in NSG mice. Statistical significance was determined by repeated measures two-way ANOVA with Sidak's multiple comparisons test and defined as $*p < 0.05$ compared to VXL + saline control. All data are shown as mean ± SEM; n = 6 for all groups.

Day	VXL + saline		VXL + MCC950 15 mg/kg	
	Mean (10^9/L)	SEM (10^9/L)	Mean (10^9/L)	SEM (10^9/L)
0	0.34	0.06	0.12	0.04
7	0.17	0.03	0.29	0.07
14	0.36	0.12	0.32	0.13
21	0.13	0.05	0.27	0.08
28	0.13	0.01	0.20	0.06

Supplementary Table 13: Systemic administration of VXL combination regimen (vincristine: V, 0.3 mg/kg; dexamethasone: X, 5 mg/kg, *L*-asparaginase: L, 1250 KU/kg) increases cumulative scores for appearance and locomotion in NSG mice that is significantly reduced by co-administration of MCC950. Statistical significance was determined by multiple unpaired t-test and defined as ∗p <0.05 compared to VXL + saline control. All data are shown as mean ± SEM; n = 6 for all groups.

Day	VXL + saline		VXL + MCC950 15 mg/kg	
	Mean	SEM	Mean	SEM
Facial grimace	0.00	0.00	0.00	0.00
Locomotion	29.00	0.00	*0.00	0.00
Behavior	0.00	0.00	0.00	0.00
Appearance	29.00	0.00	*2.50	1.45
Weight loss	0.00	0.00	0.00	0.00

Supplementary references:

1. Liem NL, Papa RA, Milross CG, Schmid MA, Tajbakhsh M, Choi S, et al. Characterization of childhood acute lymphoblastic leukemia xenograft models for the preclinical evaluation of new therapies. Blood. 2004;103(10):3905-14.
2. Lock RB, Liem N, Farnsworth ML, Milross CG, Xue C, Tajbakhsh M, et al. The nonobese diabetic/severe combined immunodeficient (NOD/SCID) mouse model of childhood acute lymphoblastic leukemia reveals intrinsic differences in biologic characteristics at diagnosis and relapse. Blood. 2002;99(11):4100-8.
3. Coll RC, Hill JR, Day CJ, Zamoshnikova A, Boucher D, Massey NL, et al. MCC950 directly targets the NLRP3 ATP-hydrolysis motif for inflammasome inhibition. Nat Chem Biol. 2019;15(6):556-9.
4. Starobova H, Monteleone M, Adolphe C, Batoon L, Sandrock CJ, Tay B, et al. Vincristine-induced peripheral neuropathy is driven by canonical NLRP3 activation and IL-1beta release. J Exp Med. 2021;218(5).
5. Szymanska B, Wilczynska-Kalak U, Kang MH, Liem NL, Carol H, Boehm I, et al. Pharmacokinetic modeling of an induction regimen for in vivo combined testing of novel drugs against pediatric acute lymphoblastic leukemia xenografts. PLoS One. 2012;7(3):e33894.
6. Carol H, Szymanska B, Evans K, Boehm I, Houghton PJ, Smith MA, et al. The anti-CD19 antibody-drug conjugate SAR3419 prevents hematolymphoid relapse postinduction therapy in preclinical models of pediatric acute lymphoblastic leukemia. Clin Cancer Res. 2013;19(7):1795-805.
7. Kang MH, Kang YH, Szymanska B, Wilczynska-Kalak U, Sheard MA, Harned TM, et al. Activity of vincristine, L-ASP, and dexamethasone against acute lymphoblastic leukemia is enhanced by the BH3-mimetic ABT-737 in vitro and in vivo. Blood. 2007;110(6):2057-66.
8. Jones L, McCalmont H, Evans K, Mayoh C, Kurmasheva RT, Billups CA, et al. Preclinical activity of the antibody-drug conjugate denintuzumab mafodotin (SGN-CD19A) against pediatric acute lymphoblastic leukemia xenografts. Pediatr Blood Cancer. 2019;66(8):e27765.
